# Step by step: towards a better understanding of the genetic architecture of Alzheimer’s disease

**DOI:** 10.1038/s41380-023-02076-1

**Published:** 2023-05-02

**Authors:** Jean-Charles Lambert, Alfredo Ramirez, Benjamin Grenier-Boley, Céline Bellenguez

**Affiliations:** 1 Univ. Lille, Inserm, CHU Lille, Institut Pasteur de Lille, U1167-RID-AGE Facteurs de risque et déterminants moléculaires des maladies liées au vieillissement, Lille, France; 2 Division of Neurogenetics and Molecular Psychiatry, Department of Psychiatry and Psychotherapy, Faculty of Medicine and University Hospital Cologne, University of Cologne, Cologne, Germany; 3 Department of Neurodegenerative diseases and Geriatric Psychiatry, University Hospital Bonn, Medical Faculty, Bonn, Germany; 4 Department of Psychiatry & Glenn Biggs Institute for Alzheimer’s and Neurodegenerative Diseases, San Antonio, TX USA; 5 German Center for Neurodegenerative Diseases (DZNE), Bonn, Germany; 6 Cluster of Excellence Cellular Stress Responses in Aging-Associated Diseases (CECAD), University of Cologne, Cologne, Germany

**Keywords:** Genetics, Neuroscience

## Abstract

Alzheimer’s disease (AD) is considered to have a large genetic component. Our knowledge of this component has progressed over the last 10 years, thanks notably to the advent of genome-wide association studies and the establishment of large consortia that make it possible to analyze hundreds of thousands of cases and controls. The characterization of dozens of chromosomal regions associated with the risk of developing AD and (in some loci) the causal genes responsible for the observed disease signal has confirmed the involvement of major pathophysiological pathways (such as amyloid precursor protein metabolism) and opened up new perspectives (such as the central role of microglia and inflammation). Furthermore, large-scale sequencing projects are starting to reveal the major impact of rare variants – even in genes like *APOE* – on the AD risk. This increasingly comprehensive knowledge is now being disseminated through translational research; in particular, the development of genetic risk/polygenic risk scores is helping to identify the subpopulations more at risk or less at risk of developing AD. Although it is difficult to assess the efforts still needed to comprehensively characterize the genetic component of AD, several lines of research can be improved or initiated. Ultimately, genetics (in combination with other biomarkers) might help to redefine the boundaries and relationships between various neurodegenerative diseases.

## Introduction

Understanding the genetic component of Alzheimer’s disease (AD) has been and still is a major research challenge. The main goal is to understand the pathophysiological mechanisms involved, and the characterization of the mutations responsible for monogenic forms of AD illustrates the scale of this challenge perfectly. The discovery of pathogenic mutations in the *APP*, *PSEN1* and *PSEN2* genes in the 1990s [1–3] led to the amyloid cascade hypothesis, which has greatly influenced the AD research field for more than three decades [4]. However, the amyloid cascade hypothesis was prompted by studies of a specific, small subset of patients (representing less than 1% of cases) and is now being called partly or wholly into question by the failure of most of the therapeutic approaches developed on this basis [5]. Even though the amyloid cascade hypothesis has been regularly modified to take account of developments in our knowledge of AD [6, 7], it now appears to be too simplistic and does not encompass the complexity and diversity of the pathophysiological processes involved in the common forms of the disease. Defining the genetic component of these common forms is one way of gaining a better understanding of the fundamental disease processes.

In 1993, the first genetic risk factor for common forms of AD was discovered: the ε4 allele of the apolipoprotein E (*APOE*) gene was found to be associated with a 3- to 4-fold increase in the AD risk [8]. A year later, it was reported that the *APOE* ε2 allele was associated with a two-fold decrease in that risk [9] – confirming the major role of the *APOE* gene in AD. Despite numerous efforts, our knowledge of the genetic component of common forms of AD did not extend much beyond *APOE* between 1993 to 2009, due to methodological and technological problems [10]. Eventually, the advent of genomic approaches (including genome-wide association studies (GWASs) and next-generation sequencing) boosted our characterization of the genetics of AD.

Here, we review the latest advances in our knowledge of the genetic landscape of AD, discuss the limitations and issues we are facing, and consider the potential consequences of these genetic findings for research on AD and related forms of dementia.

### The last decade has uncovered a new genetic landscape for AD

Following the discovery of *APOE* as a major genetic risk factor for AD, more than 350 genes were selected from 1993 to 2009 on the basis of their potential implication in pathophysiological processes, and were tested in small-scale case-control association studies [11]. *SORL1* and *ACE* are the only well-established genetic risk factors for AD [12, 13] to have emerged from this candidate gene phase. This strategy was thus mostly unsuccessful (due to methodological and technological problems) and generated highly untrustworthy and confusing information for the AD research community [10].

**Fig. 1:**
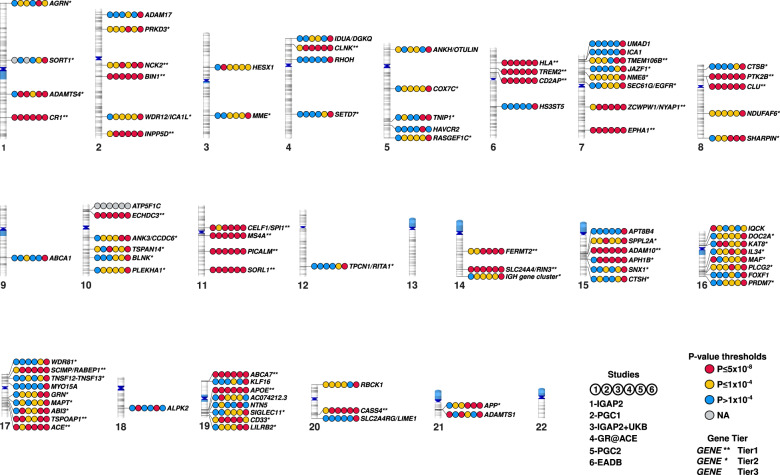
Ideogram of the 90 loci with genome-wide significance extracted from the GWAS catalog and classified as tier 1, tier 2 or tier 3. For each locus, the figure shows the *P*-value categories for the association with AD in the six main GWASs published since 2019 (IGAP2, PGC1, IGAP2 + UKB, GR@ACE, PGC2, and EADB, [20–23, 26, 27]): *P* ≤ 5 × 10^−8^, *P* ≤ 1 × 10^−4^, *P* > 1 × 10^−4^, or NA (not available). See the additional note for details of the methods.

Fortunately, AD genetics research has since greatly benefited from the advent of GWAS techniques. The results of first two seminal GWAS papers were published in 2009, with the identification of three new loci close to or within the *CLU*, *CR1* and *PICALM* genes [14, 15]. As with many multifactorial diseases, the AD GWAS field has since incorporated (i) meta-analysis methodologies for the facilitated merger of independent GWAS results [16]; (ii) larger numbers of samples (with the International Genomics of Alzheimer Project (IGAP) meta-analysis in 2013 as a milestone [17]; (iii) increasingly powerful reference population panels, which considerably improve the imputation quality of common/rare variants as well as their number for analyses; and (iv) proxy-AD cases in the UK Biobank (UKB, based on self-reports of a family history of AD-related dementia) that has increased the statistical power of the AD GWASs since the end of the 2010s [16, 18–26]. The combination of these various advances ultimately led to a recent publication by the European Alzheimer & Dementia Biobank (EADB) consortium, which reported in addition to *APOE* the association of 75 loci with AD risk, of which 42 were newly identified at that time [27]. In the GWAS catalog, there are currently 97 entries for a locus having a significant genome-wide association with the AD risk in populations of European ancestry (Supplementary Table 1). We have classified these loci as tier 1, tier 2, tier 3, or “not validated”, depending on the strength of the association in the six main GWASs performed in populations of European ancestry and published since 2019 [20–23, 26, 27] (see the additional note for a complete description of the criteria used). We classified 27, 41, 22 and 7 loci as tier 1, tier 2, tier 3 and “not validated”, respectively (Supplementary Table 1 and Fig. 1). Some of the tier 3 signals (especially those just below the genome-wide significance threshold) might be false positives, and so further validation will be required. However, it is important to bear in mind that the significance threshold is arbitrary – even though it initially corresponded to a Bonferroni correction. Hence, a number of signals of potential interest located just below this threshold have received little attention.

### GWASs in populations of non-European ancestry

The great majority of published GWASs in the field of AD were performed in populations of European ancestry, and the few performed in populations of non-European ancestry comprised limited numbers of cases and controls. Hence, comparisons are complicated by (i) the imbalance in statistical power between studies of populations of European ancestry and studies of populations of non-European-ancestry, and (ii) the risk of false positives and false negatives in small studies.

The results of the first (small) GWAS of a population of African-American ancestry were published in 2011 [28], and a GWAS comparable in size to the European studies published in 2009 was performed in 2013 [29]. The latter study found a significant, genome-wide association between a common *ABCA7* variant (which is rare in populations of European ancestry) and the AD risk. This finding emphasized the value of studying genetic diversity in populations with different ancestries, in order to capture additional information. The results of an updated GWAS with a 37% increase in the sample size (but only 826 new AD cases, in fact) were published in 2021. The study confirmed the association between the *ABCA7* variant and the AD risk, found a new signal with genome-wide significance (Supplementary Table 1), and detected nominal associations for six GWAS loci previously identified in populations of European ancestry [30].

In populations of Caribbean ancestry, few GWASs have been conducted and a small case-control study gave negative results [31]. In fact, most studies of these populations are based on linkage analyses of AD families [32]. A few case-control studies are available in central and South American countries with admixed populations [33–35]. However, many initiatives are being developed.

Lastly, although many small case-control studies of populations of East Asian ancestry have been conducted with the attempt to replicate known loci, the results of the first large GWASs of populations of East-Asian ancestry were only published in 2021: respectively four, two and two new potential loci were identified in Chinese, Japanese and Korean populations (Supplementary Table 1)[36–38].

### Next-generation sequencing: a new horizon?

Human genetic variability is primarily due to rare and very rare variants. It was soon suggested that most of the heritability of multifactorial diseases was associated with rare variants. In contrast, GWASs facilitate the analysis of variants that are frequent in the general population (those with a minor allele frequency >1%). Some genotyping chips have been specifically developed to study rare variants [39], and improved imputation panels have also facilitated this research. However, imputation tools are not effective for the vast majority of very rare variants in general and singleton variants in particular. Fortunately, the advent of next-generation sequencing and the marked fall in its cost over the last ten years have enabled the application of this technique to study rare variants in multifactorial diseases, for which large numbers of samples are required.

In 2012, the first applications of whole-exome sequencing (WES) and whole-genome sequencing (WGS)) to AD revealed that very rare/singleton mutations in the *SORL1* gene were associated with familial early-onset forms of the disease [40]. The combination of WES/WGS with imputed GWAS data identified an association between the AD risk and the R47H variant in *TREM2* (in 2013) [41, 42] and rare variants in *ABCA7* (in 2015) [43]. Since then, the associations with these rare variants in *SORL1, TREM2* and *ABCA7* have been systematically validated and extended as the number of individual sequences has grown [44]. In 2019, the Alzheimer’s Disease Sequencing Project published results of WES for 5740 cases of late-onset AD and 5096 cognitively normal controls [45]. Rare variants in several genes were found to be potentially associated with the AD risk. However, the signals observed in this study were not replicated by the Alzheimer Disease European Sequencing (ADES) consortium in the latest and most recent WES study in AD to date (with 12,652 AD cases and 8693 controls in stage one of the study) [46]. Along with *ABCA7*, *TREM2* and *SORL1*, the ADES study found AD risk signals for rare variants in two new genes (*ABCA1* and *ATP8B4*). It is noteworthy that a suggestive signal was also observed for *ADAM10*. Several important conclusions can be drawn from this work: (i) rare, damaging variants in these genes have a large effect on the AD risk; (ii) unsurprisingly, these variants are enriched in early-onset AD; (iii) all these genes with rare variants have been associated with the AD risk in GWASs of common variants. Furthermore, suggestive evidence of association was also reported with rare, damaging variants in the GWAS-identified genes *RIN3*, *CLU*, *ZCWPW1*, and *ACE* [46]. This is a remarkable convergence between signals from common variants and signals from rare variants, and so the genes concerned are likely to be of importance in understanding pathophysiological processes in AD; (iv) many of the genes with significant or suggestive signals present very rare loss-of-function mutations associated with a particular high risk of developing AD that is of particular interest at the biological level. For instance, the association observed with loss-of-function mutations in *TREM2* suggests that the missense *TREM2* R47H variant associated with an increased AD risk negatively impairs the protein’s biological function.

### We are still learning about *APOE*

As mentioned above, the association between the *APOE* gene and the AD risk was first reported in 1993 [8]. It has been estimated that at the age of 85, the lifetime risk of AD is 51% for APOE44 male carriers, 60% for APOE44 female carriers, 23% for APOE34 male carriers, and 30% for APOE34 female carriers. At the same age and without reference to the *APOE* genotype, this lifetime risk is 11% in males and 14% in females [47].

Many studies have been designed to determine which genetic factors (if any) can protect against or accentuate the risk linked to carriage of the *APOE* ε4 allele [48, 49]. WES studies have identified many novel potential associations of burden of rare variants with AD risk in individuals carrying APOE e4 alleles [50]. Some researchers reported that polygenic risk scores and genetic risk scores based on common variants associated with AD modify the disease risk and the age at onset of AD in *APOE* ε4 carriers [22, 51, 52].

In-depth analyses of the APOE locus itself have also revealed complex genetic patterns. However, these analyses are complicated by the strength of the association between *APOE* and the AD risk and the complex linkage disequilibrium in the *APOE* region. In the late 1990s, it had already been suggested that the *APOE* locus association was more complicated than a simple association between the ε2/ε3/ε4 alleles and the risk of developing AD. Several common variants in this locus have been proposed to modify *APOE* expression and promote an imbalance between APOE3 expression and APOE4 expression [53–55]. Similarly, differential regulation of *APOE* expression related to different ancestral genomic background around the locus might account for the differences in risk between populations of various ancestries [56–59]. More recently, sequencing studies have characterized two rare variants (V236E and R251G, in complete linkage disequilibrium with the ε3 and ε4 alleles, respectively) associated with a substantially lower risk of developing AD [60–62]. The V236E *APOE3* mutation has been shown to reduce APOE aggregation, enhance APOE lipidation in human brains, and reduce amyloid pathology and neuritic dystrophy in an AD-like mouse model [63]. Lastly, two copies of the *APOE3* R136S mutation were suggested to have delayed the development of AD by several decades in an individual carrying a *PSEN1* mutation [64].

### How have the results of the genetic studies influenced our knowledge of the pathophysiological processes in AD?

Integrating biological and GWAS data is essential for a better understanding of the pathophysiological processes involved. To this end, the first (and still the most popular) tool was enrichment pathway analysis. This is based on the postulate whereby a relevant pathway must be enriched in genetic risk factors for the disease in question. The results of the first attempts to apply this approach in AD were published in 2010; two studies of independent GWAS datasets highlighted the involvement of the immune system in AD for the first time [65, 66]. This observation was always confirmed as increasingly large GWASs were conducted. Enrichments in pathways involved in lipid metabolism and endocytosis also gave consistent results from one GWAS to another, while pathways directly involved in APP metabolism and Tau-related proteins became the most strongly associated pathways in the latest GWAS analyses [21, 27]. These latest findings are reassuring and underpin the major roles of the two main hallmarks of AD in the brain notably by establishing that the genetic factors implicated in common forms of AD also point to APP metabolism as a culprit. Importantly, *APP* and *ADAM10* (whose corresponding protein is responsible for α-secretase activity in the brain) are both genetic risk factors for common forms of AD [22, 27]. However, it should be borne in mind that new genetic risk factors are often first evaluated in the context of known pathways. This approach may lead to circular reasoning and thus to an artificial enrichment in specific processes.

Giving biological meaning to data from GWASs and enrichment pathway analyses has some intrinsic limitations. Firstly, a large number of human genes have never been studied in a biological context; in some cases, their biological function is still deduced simply by sequence homology. Even for genes that have been studied, information on pleiotropy and/or function in the brain is not always available. Secondly, and even though the functional variant responsible for the GWAS signal can be assigned to a non-synonymous, deleterious variant in a few cases, the functional variant is usually located in an intergenic region and probably modulates the expression of the disease-causing gene. This causal gene is usually close to the sentinel/functional variants [67]. However, the presence of several genes in a locus and/or complex linkage disequilibrium patterns that include the sentinel variant make it difficult to determine which gene is responsible for the observed association.

In order to characterize functional variants and genes, several statistical approaches have been developed. The objective is to prioritize disease-associated variants or genes in complex loci by (i) the combination of fine-mapping with methods that leverage enrichments in functional genomic annotations [26, 68]; (ii) co-localization analyses, based on the postulate whereby a GWAS signal that colocalizes with a quantitative trait locus is more likely to be functional [69, 70], and (iii) transcriptome-wide-association studies that identify gene–trait associations by integrating datasets from GWASs and gene expression studies [71]. These approaches were initially based on transcriptomic data but have been now extended to data on splicing, methylation, and protein quantitative trait loci [72–74]. Given the growing number of databases (for an entire organ or by cell type), scores of varying complexity can be used to prioritize genes of interest. However, to calculate these scores, weights have to be assigned subjectively to each level of information. In the latest AD GWAS, this approach prioritized 31 genes in the 42 novel loci associated with the AD risk [27].

Generating biological information to prioritize genes in complex GWAS loci and/or to define the genes pathophysiological roles is also challenging. As reported for other diseases in the post-GWAS era [75], our mechanistic understanding lags far behind the discovery of new AD risk loci in GWASs. Naturally, research efforts tend to shift towards “star” genes that feature damaging, non-synonymous, causal variants because they are easier to study. However, as mentioned above, most of the variants responsible for GWAS signals probably modulate the expression of the genes of interest in subtle ways; this complicates the analysis. Furthermore, the definition of phenotypes of interest in specific cell types in order to functionally characterize a gene of interest is a prerequisite that can lead to the development of hypothesis-driven approaches. Thus, the vast majority of post-GWAS functional studies have been based on aspects of the amyloid cascade hypothesis (the metabolism of APP, and the production and/or the toxicity of amyloid peptides) for a specific gene [76–78] or in systematic screens [79–82] (for a review, see ref. [83]). To a lesser extent, similar approaches have assessed the impact of GWAS-defined genes on Tau toxicity/accumulation [84–87]. Here, it is worth noting the remarkable convergence between BIN1 and Tau: (i) BIN1 modulates Tau toxicity in *Drosophila* and in mouse models [88, 89]; (ii) BIN1 interacts directly with Tau in a phosphorylation-dependent manner [90]; (iii) *BIN1* AD risk variants are associated with increased neurofibrillary tangles and higher Braak stages [88, 91, 92]; and (iv) *BIN1* AD risk variants are associated with levels of Tau/p-Tau (but not of Ab_1-42_) in cerebrospinal fluid (CSF) [93] and Tau-PET results (but not amyloid-PET results) in the brain [94, 95].

In addition to the specific case of *BIN1*, the most notable success in the genomic/post-GWAS era is undoubtedly the identification of microglia as a cornerstone in the pathophysiology of AD. In 2013, it was found that rare, non-synonymous variants in *TREM2* (a gene almost only expressed in microglia) were associated with a significant elevation of the AD risk [41, 42]. On the genetic level, the importance of the microglia has since been reinforced by the discovery of rare, non-synonymous variants in *PLCG2* and *ABI3* – both of which are particularly expressed in microglia [39]. According to the GWAS results, AD risk alleles are specifically enriched in active enhancers of monocytes, macrophages and especially microglia [96, 97]. Consequently, many AD risk loci may be mediated through gene expression or splicing in microglia [98]. Many researchers have sought to link microglia, genetic risk factors (mainly *TREM2*) and Aβ peptides together through their impact on amyloid plaque formation/compaction [99–101], toxicity, and synapse pruning [102]. In animal models, GWAS-defined genes appear to determine the microglial response to Aβ but not to Tau pathology [103, 104]. However, Aβ-activated microglia might control the seeding/spreading and accumulation of Tau pathology [105–107]; hence, the AD genetic risk would be downstream of the amyloid pathway but upstream of the Tau pathology pathway [104]. Accordingly, it has been suggested that the P522R *PLCG2* variant reduces AD progression in patients with mild cognitive impairment by mitigating Tau pathology in the presence of amyloid pathology [106].

In conclusion, the biological data produced by genomic studies have not only reinforced the roles of APP metabolism and Tau pathology in the etiology of AD but have also opened up a new field of investigation concerning the immune system in general and the microglia in particular. AD is no longer seen as a linear process defined by the amyloid cascade; in fact, it appears to be an increasingly complex phenomenon resulting from pathophysiological processes with many entry points that can trigger the disease or interact to speed up or slow-down disease progression. In view of this new paradigm for AD and the complexity suggested by the new genetic data, several new, non-exclusive hypotheses have been put forward. For example, the “cellular phase” hypothesis postulates feedback and anticipation reactions between all the various cell types in the brain [108]. The “genetically driven synaptic failure” model is based on changes in the focal adhesion pathway and the related cell signaling [83].

### Although much progress has been made, how many genetic factors have yet to be identified?

Between 2009 and 2022, the number of risk loci for AD (apart from *APOE*) rose from 3 to more than 75. These discoveries have impacted our knowledge of pathophysiological processes, which is starting to be used in translational research on potential diagnostic/prognostic tools. One can legitimately wonder (i) how many genetic risk factors remain to be identified and (ii) how valuable will be a polygenic risk score (PRS). To that end, some researchers have tried to estimate the “missing heritability” in AD.

In fact, the estimates of AD’s heritability vary greatly from one study to another. The highest values have been provided by twin studies (from 48% to 79% on the liability scale, denoted h^2^_twins_). Intermediate values have been obtained in analyses of individual-level GWAS data from unrelated individuals (from 24% to 55%, denoted h^2^_SNP_). Lastly, the lowest estimates were based on summary statistics from GWASs (from 2% to 42%, denoted h^2^_summary_)(Supplementary table 2). These differences are not unexpected, since heritability is a population-dependent measure. Differences between twin studies can also be explained by the rather small sample sizes considered, which lead to large confidence intervals for the estimates. However, all the h^2^_twins_ estimates are quite broad, and a meta-analysis of twin studies estimated the heritability of dementia in AD to be 62.7% [109]. As has been observed for many complex diseases or traits, intermediate heritability values have been estimated in analyses of individual-level GWAS data from unrelated individuals. There are several explanations for this observation, including poor tagging of causal variants in GWAS data (for example rare causal variants), or the over-estimation of heritability in twin studies due to a common environment, gene-gene interactions, or gene-environment interactions [110–112]. More generally, the genomic heritabilities h^2^_SNP_ and h^2^_summary_ are specific to the variant set considered. Filtering on the minor allele frequency is usual, sex chromosomes are excluded, and h^2^_summary_ is computed after removing the major histocompatibility complex region (which contains a genetic risk factor for AD) and loci with large effects (such as *APOE*); this leads to underestimation of the heritability. Furthermore, the restricted maximum likelihood method (used to compute h^2^_SNP_ for AD) reportedly underestimates the heritability of binary traits in case-control studies, and, like the linkage disequilibrium score regression (LDSC) approach (used to compute h^2^_summary_) [113], can provide biased estimates in the presence of strong non-genetic risk factors (such as age, for AD) [114, 115]. Variability in h^2^_SNP_ and h^2^_summary_ is due in part to the difference in the prevalence values considered (from 2% to 33%). The prevalence varies greatly with age but also with sex and has a large impact on the estimation of heritability. Hence, an increase in the prevalence from 2% to 33% means that h^2^_SNP_ rises from 24% to 55% [116, 117]. The h^2^_summary_ values are lower than the h^2^_SNP_ values and range from 2% (with a prevalence of 5%) to 25% (with a prevalence of 17%), although one of the studies considered had a small sample size and estimated an outlying value of h^2^_summary_ of 42%. However, h^2^_summary_ is commonly computed with the LDSC approach, and considering linkage disequilibrium in an external reference panel rather than in the study sample, which biases the estimates [118, 119]. Furthermore, LDSC reportedly underestimates heritability when a major mutation (such as *APOE*) explains a high proportion of the heritability [120]. h^2^_summary_ is most often computed from GWAS meta-analysis results and tends to decrease with the size of the meta-analysis sample. This phenomenon has been observed for other diseases and traits and might be due to inter-study heterogeneity with regard to LD and characteristics of the study populations (such as age and *APOE* status), the accuracy of diagnoses, and the use of unscreened controls in the largest AD meta-analyses [119, 121–123]. In particular, the largest AD meta-analyses included some proxy-AD cases and thus assessed the genetics of AD and related dementias rather than AD alone. Furthermore, some studies do not take account of proxy-cases correctly when computing heritability, which leads to underestimated values [117]. Lastly, varying levels of ascertainment in meta-analyzed studies can also result in underestimation if it is not appropriately accounted for [124].

Overall, estimating the heritability of AD remains methodologically challenging. From a conceptual point of view, many of the inherent assumptions in the statistical models used to estimate heritability are not appropriate for a disease as complex as AD. This raises the question of whether estimates of the “missing heritability” are meaningful [111, 125].

### How can we improve our knowledge of the genetics of AD?

The difficulty of understanding the genetics of AD is clearly illustrated by the fact that some researchers suggest that AD is an oligogenic disease involving around 100 common causal variants [120], while others favor a polygenic model with up to 11,000 common causative variants [126]. Consequently, it is reasonable to consider prudently that new loci have yet to be identified and that known loci require further characterization in AD.

Of course, it will always be necessary to increase the sample size (whether of European ancestry or multiple ancestries) in classical GWASs and sequencing analyses, in order to capture the genetic information carried respectively by common and rare variants. It will also be necessary to analyze all the types of variations in the genome. Structural variants (SVs i.e. changes larger than 50 bp) have been poorly studied in AD. SVs and (especially) copy number variations are major sources of genomic variation; although two individuals may differ genetically by 0.1% when considering single nucleotide variations, the difference increases to 1.5% when SVs are also taken into account [127–129]. However, apart from gene duplications leading to monogenic forms of neurodegenerative diseases [130–132], this field that has been poorly studied in common AD and the few available studies of GWAS addressing SV association with AD data lack consistency [133–137]; this might be attributable to various technical biases (e.g. different genotyping and sequencing platforms), batch effects, and a lack of statistical power. Nevertheless, methodological and technical progress (especially long-read sequencing) will probably make SVs easier to detect [138, 139].

Along with the ability to fully capture the variability in the human genome, it is also important to exploit this knowledge through a wide range of approaches to characterize the genetic component of AD. Fine-mapping approaches are needed to better understand the real importance of a given gene/locus: the risk conferred by a gene/locus can be underestimated if one is unaware of the existence of several independent causal variants, as observed for *BIN1* [26]. Heterogeneous or even hidden genetic signals (e.g. those that depend on the *APOE* genotype, sex, or early versus late onset) can also be assessed in interaction and stratification analyses. At another level of complexity, pangenomic searches for gene-gene or gene-environment interactions can be developed [140–142]. These approaches nevertheless require significant computing power and can generate false positives through multiple statistical testing. For gene-environment interactions more specifically, the question of statistical power arises because the longitudinal studies performed to date (e.g. the CHARGE consortium) included some tens of thousands of individuals. However, this limitation is being lifted by the creation of large biobanks (such as the UKB) in which the number of cases diagnosed will increase as the study population ages. Lastly, the implementation of increasingly large GWASs of many AD-related endophenotypes [93, 143–146] and the GWAS datasets’ integration with other “omics” databases (e.g. systems biology) should make it possible to characterize key elements of AD genetics and related pathways. Importantly, the move towards systematic, detailed integration of the available data will make it difficult to validate results obtained independently. It will therefore become essential to demonstrate the biological relevance of genetic results (some of which will be generated by artificial intelligence) in appropriate cellular and/or animal models.

Lastly, many methodological issues related to heterogeneity in the generation of summary statistics can impact the results of GWASs and complicate comparisons and subsequent analyses. For example, analyses of proxy-AD cases require a correction factor, which can differ across studies, different covariates are used to adjust statistical models, and different imputation panels are used.

**Fig. 2:**
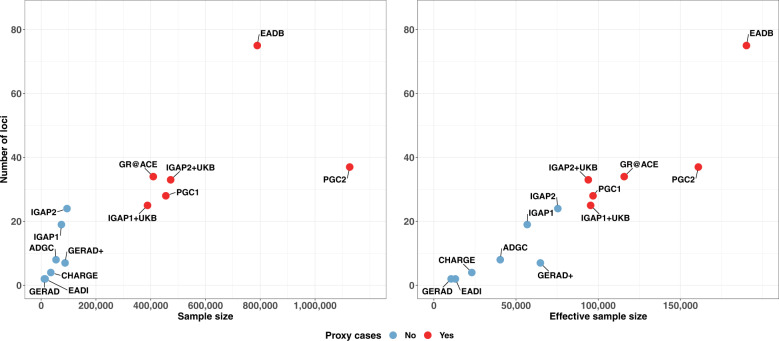
Number of loci identified in the different GWASs. Number of loci identified as a function of the sample size (left) or the effective sample size (right) (cases and controls) in the main GWASs published since 2009 (EADI, GERAD, CHARGE, GERAD+, ADGC, IGAP1, IGAP1 + UKB (2017), IGAP1 + UKB (2018), PGC1, IGAP2, IGAP2 + UKB, GR@ACE, PGC2, and EADB [14–27]. The colors indicate the presence or absence of proxy cases in the GWAS. The effective sample size was computed per study included in the meta-analyses, and then summed across studies [124]. The effective sample size for the proxy UKB study was computed by dividing the raw number of proxy cases and proxy controls by four [24, 117].

Since the IGAP’s results were published, AD GWASs have been carried out by the meta-analysis of shared and (potential heterogenous) summary statistics and many findings might be driven by the IGAP summary statistics. Further, the number of controls has increased more quickly than the number of patients, following access to the very large population-based biobanks; this has led to stagnation in the number of novel loci characterized (Fig. 2).

The EADB was set up against this background. The biobank’s main objective was to double the number of new, clinically diagnosed cases of AD available for analysis; this probably explains why the number of detected loci increased markedly (Fig. 2). Furthermore, the EADB shared raw data from the existing European GWASs, which enables the data to be checked for potential sample overlaps. It also facilitates the use of the same imputation panel (TOPMed), the application of the same quality control procedures, and the implementation of similar statistical models for homogeneous summary statistics prior to meta-analysis. Overall, sharing raw data improves and speeds up GWAS meta-analysis. Unfortunately, although the USA has a remarkable data sharing policy to be commended and encouraged, the European Union’s General Data Protection Regulation (a legitimate effort to protect individual data) restricts data sharing outside Europe (and even into Europe).

### Will genetics impact the definition of AD and related dementias?

In addition to the inherent methodological issues of GWASs, the diagnosis of AD can also be questioned and debated. The definition of around 40,000 to 50,000 proxy-AD cases in the UKB can be criticized because it is based on self-reports of a family history of AD dementia. Although the large number of proxy-AD cases increases the statistical power of the AD GWAS, and the genetic correlation between AD and proxy-AD is high (above 0.65) [20, 23, 25, 117], this “virtual diagnosis” might lead to the inclusion of misdiagnosed individuals. In addition, even when studying clinically diagnosed cases, it is estimated that around 10–20% of diagnoses are incorrect. These points have prompted some experts to even consider that the detection of the same genetic signals for several neurodegenerative diseases could be artefactual; because of these misdiagnoses and the size of the AD GWASs, these GWASs were also statistically powerful enough to detect signals corresponding to the pathologies associated with these misdiagnoses.

To circumvent this problem, the selection of individuals on the basis of post-mortem pathology assessments is obviously the best option for establishing a definitive diagnosis. However, this approach is still strongly limited by the lack of access to large collections of brain samples – especially for age-matched controls [91, 147, 148]. Another option would be to use biomarker profiles to distinguish between AD cases, controls, and individuals with other neurodegenerative diseases. However, generalization of the recently developed amyloid/Tau/neurodegeneration (A/T/N) classification is limited by the cost of CSF biomarker assays and imaging [149]. Furthermore, the biomarker field is evolving rapidly, with the discovery of novel targets (e.g. p-Tau 217 and p-Tau 231) with potentially high diagnostic/prognostic value [150, 151]. These biomarkers might lead to a refinement of the diagnostic profile for AD and related disorders and therefore the corresponding genetic studies. However, these approaches might also lead to the over-selection of cases that are not representative of AD and the latter’s complex relationship with the neurodegenerative landscape in real life.

Indeed, the postulate whereby detection of the same genetic signal in two different neurodegenerative diseases results from misdiagnosis is probably too simplistic and does not fully take account of important data from genetics and, above all, brain pathology markers. Firstly, in GWASs of clinical frontotemporal dementia, *TMEM106B* variants achieved only modest p-values and odds ratios for the behavioral subtype of frontotemporal dementia [152], and the associations were dependent on *GRN* mutations [153]. It is unlikely that this clinical subpopulation alone could drive a genome-wide, significant signal in an AD GWAS. Secondly, and even though a locus can be common to several diseases, it appears that signals (and thus functional variants) can differ – as observed for the *IDUA* locus in AD and in Parkinson’s disease. This may indicate that a common locus does not necessarily have the same pathological consequences in different neurodegenerative diseases. Thirdly, patients with AD often have other (concomitant) neurological diseases, which are potential “partners in crime” [154]. For instance, *GRN* and *TMEM106B* are reportedly involved in defective endosome/lysosome trafficking/function – a defect that is also observed in AD [155, 156]. *GRN* protects against amyloid-β deposition and toxicity in AD mouse models [157]. This is also illustrated by the association between *BIN1* and the risk of developing Lewy body dementia, as observed in a large sample of autopsy-confirmed and clinically probable cases [92]. This *BIN1* signal (like that observed in AD) was significantly associated with increased neurofibrillary tangles, as also observed in AD brains [90, 91]. Given the many observations linking *BIN1* to Tau-related endophenotypes [93, 94] and pathological processes [89, 90], the identical genetic signal for *BIN1* in two diseases pathologies suggest that similar Tau-related pathological processes are operating.

In general, one can argue that the initial and/or subsequent localization and development of concomitant diseases can trigger or favor AD. In other words, the interplay between pathophysiological processes in different pathologies might prompt the detection of a common genetic locus. The presence of common causal variants in the same gene might indicate a shared pathological role, whereas the presence of different causal variants in the said gene might indicate specific mechanisms or cell-type-specific expression. Common genetic risk factors in neurodegenerative diseases might have thus several important implications and do not necessarily reflect misdiagnosis in GWASs. In combination with other biomarkers, genetic markers could perhaps redefine the boundaries and relationships between neurodegenerative diseases (i.e. a hypothetical continuum). Hence, the inclusion of this information may be particularly useful for better defining AD and concomitant diseases in real life. For example, the development of PRSs might help to integrate this heterogeneity into pathophysiological processes for a given case – even when this case has been diagnosed as AD. This may be of particular importance when developing drugs potentially targeting shared genes/pathways and the development of an efficient precision medicine.

### Development of a tool for the diagnosis and prognosis of the common forms of AD

Translating new genetic knowledge into clinical practice (e.g. to identify individuals at risk of progression to dementia) is challenging, for many reasons. Researchers have designed PRSs that reflect an individual’s overall genetic burden for a given disease [158–161]. The PRS provides a cumulative effect score summarizing the small information distributed across the individual susceptibility variants. To construct a PRS, an effect estimate has to be assigned to each variant included in the PRS; this estimate is obtained from the summary statistics derived from large GWASs and meta-analyses thereof [162]. This large GWAS dataset is considered to be the “training” dataset from which the effect estimators are obtained and used to build an informative PRS using an independent dataset called validation or test dataset. In this regard, the constant increase in sample size in GWASs has contributed significantly to the identification of genuine risk variants for diseases and has improved the effect estimates for each risk variant. All this information helps to better calibrate PRSs [163] for the assessment of at-risk individuals and the scores’ translation into strategies for better diagnosis and (eventually) early intervention and prevention. PRS methodologies have been improved by the availability of genomic information on linkage disequilibrium dependencies between variants; this enables better definition of a single tagging variant for a defined locus and avoids redundant information and falsely inflated results [160, 164].

The potential value of PRSs in the context of AD has been emphasized by studies in which an AD-derived PRS was associated with the clinical diagnosis [165], cortical thickness [166], memory, hippocampal volume, cognitive decline [167], disease progression [168], and post-mortem confirmed cases [169]. However not everything has been “hunky-dory” with PRS because results from studies using an AD-derived PRS, incorporating all or part of the GWAS signals, have been inconclusive with regard to disease progression and clinical diagnosis [170, 171]. Thus, these contradictory results cast some doubts on the strategy used to compute the PRS. Consequently, several studies have focused on determining the best overall way to compute a PRS (for a more detailed review see [172]).

The term “PRS” is used liberally to refer to scores that include various number of SNPs. Current methods for PRS computation are designed to reduce the signal-to-noise ratio by selecting a small number of the most informative SNPs [173]. This specific selection of SNPs in the final model follows different selection criteria such as significance thresholds or a priori selection by researchers. This selection generally includes only common SNPs (minor allele frequency 5%) using a model that assumes only additive effects for these variants, and do not account for additional components like SNPxSNP interactions and dominance [174, 175]. Besides, rare variants (minor allele frequency <1%) or copy number variations are normally left out of a PRS as they are not included in the genetic data derived from GWAS. Thus, these inclusion criteria may restrict the genomic information included in the model leading to the inability of PRS to completely capture the genomic landscape of the selected trait [176, 177]. Other important aspects of PRS calculation are subject to debate: the best way to model *APOE* within a PRS, the p-value threshold for including SNPs, and the comparison of PRSs for independent cohorts [173, 178, 179]. As mentioned above, and although it is clear that several genes are involved in the AD process, there is still debate as to whether AD is polygenic or oligogenic [120, 165]. Resolving these issues will have a major impact on the strategy used to build a PRS in AD. Interestingly, a recent GWAS of human height (a strongly polygenic and heterogeneous trait) in over 5 million participants appeared to reach a plateau for the identification of genetic variants [180]. The investigators concluded that substantially increasing the sample size of GWAS might largely resolve the heritability attributed to common variation by identifying a finite set of SNPs [180].

While most of the debate concerning PRS has focused on selecting SNPs from a single summary statistic dataset, less attention has been paid to the fact that AD is a complex, heterogeneous phenotype [181]. As previously indicated, the pathophysiological processes in AD are diverse, and not all will be present and operating at the same time in a given patient. Hence, two patients with AD will present different subsets of the pathophysiological processes. Moreover, the various pathogenic processes and endophenotypes associated with AD might be driven by different genetic variants – ones that are not necessarily involved in the susceptibility to AD identified by case-control GWAS. In support of this hypothesis, GWASs using CSF levels of the hallmark biomarkers of AD (i.e. amyloid beta and tau) have identified a handful of genetic variants involved also in the susceptibility to AD [93, 182]. These studies also suggest that the genetics driving each of the biomarker’s levels in CSF are independent of each other – except for the *APOE* locus. On the same lines, GWASs of cognitive performance in more than 300,000 cognitively healthy individuals also reported a handful of genome-wide significant signals in regions overlapping with loci responsible for susceptibility to AD [183]. This finding suggests that the genetic factors identified in case-control GWASs will only partly explain the variance observed in the AD phenotype. Hence, a simple PRS derived from case-control summary statistics might not explain the total genetic risk in an AD patient. As discussed above, some of the genetic drivers of AD are probably also involved in other types of dementia. Accordingly, a PRSs encompassing the genome-wide significant SNPs identified by the EADB consortium was consistently associated with progression to dementia in various cohorts of patients with mild cognitive impairment (prodromal dementia) and cognitively healthy individuals [27]. These findings underscore the contribution of the identified genetic variants to the progression of cognitive decline along the continuum of AD and may be also to related diseases, though this latter suggestion needs further research.

In conclusion, while PRS has been seen as a path to personalized medicine, the use of PRS in medicine is not exempt from limitations. PRS informs about the probability and not a destiny of a person based on the genetic burden of a particular disease. Hence, whether or not a PRS translates into pathology will not be reported by the PRS itself but from dynamic markers showing the ongoing pathology. This, however, also states the question of whether using PRS for age-related diseases in young populations may have any impact on prevention. However, our PRSs will become increasingly accurate as our knowledge of the genetics of susceptibility to AD improves. A PRS that provides information on the genetics of susceptibility to AD and on the related underlying endophenotypes would be especially useful. Likewise, strategies based on hazard ratios (rather than risk scores) might also improve our predictive ability and could ultimately lead to the “holy grail”, i.e., a score for identifying cognitively healthy young individuals at risk of developing dementia. Identification of the pathways affected in each individual at risk would open up many opportunities, such as the development of personalized prevention strategies.

## Conclusion

The AD genetic community has a clear responsibility for accelerating and fostering the generation of biological and clinically relevant results. The characterization of the genetics of multifactorial diseases like AD has a major impact on the research in the fields and therefore the development of treatments. By way of an example, human genetic evidence supported two-thirds of the drugs approved by the US Food and Drug Administration in 2021 [184]. In view of this major responsibility, geneticists must ask themselves essential questions about their research practices and the resulting publications. This implies an appropriate understanding of the various biases in the results, how these biases should be taken into account, and how the results should be published. Geneticists must also be prepared to correct their mistakes, given the inherent risk of generating false positives in genetic studies.

Over the last ten years, we have made remarkable progress in understanding the genetics of AD but considerable additional efforts are still needed. Nevertheless, one can assume that the most obvious genetic factors/loci have already been identified; the remaining research efforts and investments will have to define new factors/loci with restricted impact – even though rare variants can still have a moderate impact on the AD risk. Regardless of how much of the genetic variability in AD remains to be discovered, knowledge of AD genetics has already had a considerable impact on (i) our understanding of the pathophysiology of AD (e.g. the central role of microglia), (ii) the definition of sub-populations at risk, and (iii) the interplay between AD and other neurodegenerative diseases. These genetics-related advances are especially important because few genetic risk factors have been studied in detail after their initial identification as being associated with the AD risk. We can therefore legitimately be optimistic and assume that the growth in post-GWAS research (such as the MODEL-AD project [185]) will enable to make rapid progress in the years to come.

### Supplementary information

supplementary tables 1-2

supplmentary material
