# Supplementary material for: Step by step: towards a better understanding of the genetic architecture of Alzheimer’s disease
Source: Mol Psychiatry. 2023 May 2;28(7):2716–27. doi: 10.1038/s41380-023-02076-1 (PMC10615767; doi:10.1038/s41380-023-02076-1)
Supplement: Supplementary file 2 — supplmentary material [file 41380_2023_2076_MOESM2_ESM.pdf]

Additional Note: Loci definition (Figure 1 and Supplementary Table 1)

The GWAS Catalog was downloaded on 2022-06-15 [1]. We kept only variants with a genome-wide significant signal ($P <= 5x10^{-8}$) for one of those “MAPPED_TRAIT”: "late-onset Alzheimers disease", "Alzheimer disease", "Alzheimer disease, family history of Alzheimer’s disease" or "family history of Alzheimer’s disease", leading to 837 signals for 510 unique variants of interest. We attributed to each variant the minimum P-value across all signals reported for this variant in the GWAS catalog. Based on those variants of interest, genomic loci and lead variants were then defined by FUMA using the default parameters, and including the MHC region [2].

16 variants of interest were not available in FUMA using the 1000G reference panel. Those located less than 250 kb of a lead variant were assigned to that variant’s locus. Otherwise, a new locus was created, with the variant of interest as the new lead variant. The *PTK2B* locus was also manually added, as it has been shown to be statistically independent from the *CLU* locus [3, 4].

We do not report a lead variant for the *APOE* locus.

We report the result of each lead variant in each of the 6 latest and largest AD GWAS meta-analyses: IGAP2 [5], PGC1 [6], IGAP2+UKB [7], GR@ACE [8], PGC2 [9] and EADB [4]. Those results were extracted from the genome-wide summary statistics provided by the authors, which might not include all the Stages, or all the datasets, included in the paper. Those results might thus be different from the ones reported by the GWAS catalog, which are extracted from the paper.

In addition, for each locus except *APOE*, we report:

a) the top tag variant in each of the 6 GWAS summary statistics. A tag variant is defined as a variant with $r^2 >= 0.6$ with the lead variant, according to the linkage disequilibrium (LD) data provided by TopLD for the European TOPMed panel [10]. The top tag variant is then defined as the tag variant with the minimum P-value across all tag variants;

b) the top variant among the 510 variants of interest extracted from the GWAS catalog for the 6 GWAS (top GC variant). Note that i) for IGAP2, the GWAS catalog only reports the results for the new loci; ii) for IGAP2+UKB, the GWAS catalog reports results of the discovery stage, only for loci that have more than one signal.

c) the top variant in each of the 6 GWAS summary statistics, using the start and end of the loci provided by FUMA (independently of LD) (top locus variant). This information is thus not available for the loci that were manually added.

For variants selected in b) and c), we report linkage disequilibrium measured by the r^2 statistic provided in the European TOPMed panel by TopLD (only available if $r^2>0.2$), and in the 1000G EUR panel by LdlinkR [11].

The pipeline required to lift variants position and alleles from the GRCh37 assembly to the GRCh38 assembly, and vice-versa. For that, we used the UCSC liftover software (https://genome.ucsc.edu/cgi-bin/hgLiftOver), the liftoverVcf tool from Picard (http://broadinstitute.github.io/picard), or a custom pipeline [4].

The P-value of a locus (reported in Figure 1) is set as the minimum of the P-values of the lead variant, the top tag variant, the top GC variant and the top locus variant. Loci were then divided into five groups:

- "Tier 1": the lead variant is genome-wide significant in EADB and PGC2, and suggestive in IGAP2 ($P <= 1x10^{-4}$);
- "Tier 2": the lead variant is genome-wide significant in EADB or PGC2, and suggestive in PGC2 or EADB, respectively;
- "Tier 3": loci not classified as "Tier 1", "Tier 2", "Not validated", or "Other";
- "Non validated": the lead variant is not at least suggestive in both EADB and PGC2;
- "Other": loci that have been identified in multi-ethnic studies, in samples of non-European ancestry, in stratified analyses, for endophenotypes, or other specific studies.

The chromosome ideograms were obtained from Genome Decoration Page (https://www.ncbi.nlm.nih.gov/genome/tools/gdp).

1. Buniello A, MacArthur JAL, Cerezo M, Harris LW, Hayhurst J, Malangone C, et al. The NHGRI-EBI GWAS Catalog of published genome-wide association studies, targeted arrays and summary statistics 2019. Nucleic Acids Res. 2019;47:D1005–D1012.
2. Watanabe K, Taskesen E, van Bochoven A, Posthuma D. Functional mapping and annotation of genetic associations with FUMA. Nat Commun. 2017;8:1826.
3. Lambert JC, Ibrahim-Verbaas CA, Harold D, Naj AC, Sims R, Bellenguez C, et al. Meta-analysis of 74,046 individuals identifies 11 new susceptibility loci for Alzheimer's disease. Nat Genet. 2013;45:1452–1458.
4. Bellenguez C, Küçükali F, Jansen IE, Kleineidam L, Moreno-Grau S, Amin N, et al. New insights into the genetic etiology of Alzheimer's disease and related dementias. Nat Genet. 2022;54:412–436.
5. Kunkle BW, Grenier-Boley B, Sims R, Bis JC, Damotte V, Naj AC, et al. Genetic meta-analysis of diagnosed Alzheimer's disease identifies new risk loci and implicates Aβ, tau, immunity and lipid processing. Nat Genet. 2019;51:414–430.
6. Jansen IE, Savage JE, Watanabe K, Bryois J, Williams DM, Steinberg S, et al. Genome-wide meta-analysis identifies new loci and functional pathways influencing Alzheimer's disease risk. Nat Genet. 2019;51:404–413.
7. Schwartzentruber J, Cooper S, Liu JZ, Barrio-Hernandez I, Bello E, Kumasaka N, et al. Genome-wide meta-analysis, fine-mapping and integrative prioritization implicate new Alzheimer's disease risk genes. Nat Genet. 2021;53:392–402.
8. de Rojas I, Moreno-Grau S, Tesi N, Grenier-Boley B, Andrade V, Jansen IE, et al. Common variants in Alzheimer's disease and risk stratification by polygenic risk scores. Nat Commun. 2021;12:3417.
9. Wightman DP, Jansen IE, Savage JE, Shadrin AA, Bahrami S, Holland D, et al. A genome-wide association study with 1,126,563 individuals identifies new risk loci for Alzheimer's disease. Nat Genet. 2021;53:1276–1282.
10. Huang L, Rosen JD, Sun Q, Chen J, Wheeler MM, Zhou Y, et al. TOP-LD: A tool to explore linkage disequilibrium with TOPMed whole-genome sequence data. Am J Hum Genet. 2022;109:1175–1181.
11. Myers TA, Chanock SJ, Machiela MJ. LDlinkR: An R Package for Rapidly Calculating Linkage Disequilibrium Statistics in Diverse Populations. Front Genet. 2020;11:157.
